# The Influence of Active Removal from Working Memory on Serial Dependence

**DOI:** 10.5334/joc.222

**Published:** 2022-05-24

**Authors:** Jiangang Shan, Bradley R. Postle

**Affiliations:** 1 Department of Psychology, University of Wisconsin-Madison, Madison, US; 2 Department of Psychiatry, University of Wisconsin-Madison, Madison, US

**Keywords:** Working memory, Attention, Cognitive Control

## Abstract

Flexible control of the contents of working memory (WM) includes removing no-longer-relevant information. Although simply withdrawing attention offers a “passive” mechanism, empirical findings suggest that it is also possible to actively remove information from WM. In this Registered Report we tested evidence that the bias (serial dependence) that an item exerts on the subsequent trial will be opposite in sign—attraction vs. repulsion — depending on whether it was passively or actively removed, respectively. A repulsive bias would be consistent with a specific mechanism for active removal: a rapid adaptation-like modification of perceptual circuitry. In a preliminary study, trials of two types were administered in pairs, multi-item WM followed by 1-item delayed recall, and we evaluated serial dependence of the latter on items from the former. In the first trial of each pair, two memoranda were presented, then one was designated irrelevant, then a third memorandum was presented. The critical manipulation was whether the third item was presented at the same location as the now “irrelevant memory item” (IMI). Overlap between the two should prompt the active removal of the IMI, whereas nonoverlap might prompt just the withdrawal of attention. Whereas the IMI exerted the expected attractive bias on 1-item recall in the *no-overlap* condition, we found an (unexpected) repulsive bias in the *overlap* condition. Because repulsive biases have been attributed to the adaptation-like modification of perceptual circuitry, replication of this result in this Registered Report would provide independent evidence for this mechanism for active removal from WM. Interpretation of the Stage 2 results are complicated by the fact that the approved Registered Report, carried out online, generated data that failed to meet a basic sanity check, and were therefore uninterpretable. Consequently, a follow-up lab-based experiment using procedures similar to the Registered Report generated results consistent with the hypothesis of principal theoretical interest: The IMI in the *overlap* condition exerted a repulsive bias on the subsequent trial.

## Introduction

One way that the cognitive system compensates for the capacity limitations of working memory (WM) is via the flexible updating of the contents of WM and the control of priority among those contents. For example, a memory item that is needed to guide current behavior is prioritized and represented in a different state relative to items that are not immediately relevant, but might be needed in the future (Lewis-Peacock & Postle, 2012; Rose et al., 2016; van Loon et al., 2018; Yu, Teng, & Postle, 2020). Additionally, when a memory item loses relevance, it can be actively removed from WM (Fulvio & Postle, 2020). The focus of this Registered Report is to assess the mechanism whereby an item in WM can be strategically removed, and how this may differ from the passive loss that is assumed to occur, for example, at the end of a trial.

The extensive history of the study of proactive interference in memory, including WM, documents the fact that subjects do not routinely employ active removal, or at least not effectively. For example, in a WM task using a recognition procedure in which subjects memorize a set of letters and then, after a delay period, judge whether the probe presented at the end of the trial was in the memory set, “recent-negative” probes not in the memory set of the current trial but that were in the memory set of the previous trial are rejected with lower accuracy and longer RTs than “nonrecent-negative” probes (Monsell, 1978). Whereas infrequent recent-negative probes recruit reactive control (Braver, 2012; Burgess & Braver, 2010) that is supported by phasic activity in inferior prefrontal cortex (PFC; D’Esposito et al., 1999; Feredoes et al., 2006), high levels of recent-negative probes recruit dorsal PFC-supported proactive control (Braver, 2012; Burgess & Braver, 2010). Importantly, however, these examples of control are not necessarily related to the removal of information: proactive control could prompt stronger encoding of trial-specific context, whereas reactive control would influence the recognition decision (resolution of the conflict between the familiarity of the recent negative item versus the recollection of the memory set (Feredoes & Postle, 2010)).

Visual WM tasks using a recall procedure (a.k.a. delayed estimation), also reveal evidence of less-than-complete loss of information from trial to trial. On these tasks, subjects first encode the critical feature of the sample stimulus (e.g., the orientation of a Gabor patch) and then, after a delay period, they report that feature (e.g., recreate the remembered orientation with a response dial). On this type of WM task, the sample shown on each trial is typically chosen at random, and so on these tasks it is also assumed that the representation of the sample is “dropped” from WM once the response is made. Nonetheless, a common observation on this type of task is that the recall report on the current trial is attracted toward the value of the memory item from the previous trial (e.g., Bliss et al., 2017; Fischer & Whitney, 2014). (E.g., if the orientation of the to-be-recalled sample on the current trial is 90°, and the orientation of the sample on the previous trial had been 120°, there is a tendency for recall to be biased toward 120° (i.e., a recall value of 92° is more likely than a recall value of 88°)). This attractive influence of the previous trial is called serial dependence, and it indicates that stimulus information from a trial is typically not totally removed at the end of the trial. Indeed, two recent studies have found direct evidence for this. With electroencephalography (EEG) data collected during delayed recall for orientation, Bae and Luck (2019) were able to decode the orientation of the previous trial’s sample after the onset of the current trial’s sample. For delayed recall of location, reactivation of an activity-silent representation of the previous trial’s sample location was observed near the end of the intertrial-interval (ITI) in PFC in nonhuman primates, as was a similar reactivation in whole-scalp EEG in humans. These effects were modelled as the consequence of the filtering of a “nonspecific anticipatory signal” by a residual trace of the representation of the sample “imprinted in neuronal synapses as a latent activity-silent trace” (Barbosa, Stein et al., 2020). The dynamics of the bump-attractor model of Barbosa, Stein et al. (2020) explicitly capture our intuition of what it means to assume a passive loss of information from WM (a.k.a. decay-based forgetting): it is modeled by simply removing activation from the elements that represent the information that had been held during that trial, and the bump of elevated activity recedes to baseline (Barbosa, Stein et al., 2020). We note that the same dynamics have also been assumed for the within-trial loss of information from WM, such as during a task when new information leads to the “reallocation” of resources away from a newly irrelevant item (Chatham & Badre, 2013).

In contrast to the idea of a “passive loss” of information from WM, as reviewed up to this point, there are also theoretical reasons to postulate, and empirical evidence for, an active removal mechanism. These derive from tasks in which more than one item is held in WM simultaneously, and so competition is occurring between stimulus representations being held simultaneously (rather than between items from different trials). In visual WM, it is well-established that performance declines as a monotonic function of load (e.g., Ma et al., 2014). Performance improves, however, when a retrodictive cue (“retrocue”) that appears during the delay indicates which item from the memory set is the one that will be tested at the end of the trial (e.g., Lepsien & Nobre, 2006). So too does the strength of the neural representation of the retrocued item (Sprague et al., 2016). Behavioral evidence from a slightly more complicated task — dual serial retrocuing (DSR) — suggests that this retrocuing benefit is due to the active removal of the uncued item from WM. In DSR, there are two memory probes, each one preceded by a retrocue. After the offset of the trial’s two sample stimuli, the first retrocue indicates which item will be tested by the first probe. However, the uncued item can’t yet be dropped, because a second retrocue will then indicate which of the two will be tested by the second memory probe, and both memory items are equally likely candidates for this second retrocue. Thus, the DSR requires subjects to control the priority status of items held in WM, with the first retrocue designating which item is the prioritized memory item (PMI) and which the unprioritized memory item (UMI). Although functional magnetic resonance imaging (fMRI) and EEG studies indicate that evidence for an active representation of the UMI can decline to baseline levels (LaRocque et al., 2013; Lewis-Peacock & Postle, 2012), or transform into a different representational format (Wan et al., 2020; Yu, Teng, & Postle, 2020), a pulse of TMS during the delay following the first retrocue has two effects: it evokes a brief reactivation of the representation of the UMI; and it boosts the rate of false alarms when the UMI is used as a recognition probe (Rose et al., 2016). The evidence for an active removal process comes from the absence of comparable reactivation effects when TMS is delivered following the second retrocue on DSR trials (Rose et al., 2016), or following the sole retrocue on single-retrocue trials (Fulvio & Postle, 2020).

What might be the mechanism that implements the active removal of information from WM? Different theoretical frameworks provide different answers to this question. In a bump-attractor model, removal would be accomplished by a nonspecific burst of activation broad and strong enough to swamp the stimulus-representing bump and to saturate the residual synaptic trace. In the interference model (Oberauer & Lin, 2017), active removal of an item from WM is accomplished by breaking the association between an item and its context (Lewis-Peacock et al., 2018). A third, intuitive, possibility for active removal is the suppression of the neural representation of the to-be-removed item. Testing an explicit mechanism whereby this third mechanism might be implemented is the motivation for this Registered Report, which is a replication of an experiment whose results gave rise to this idea.

### Preliminary Study

**Figure 1:**
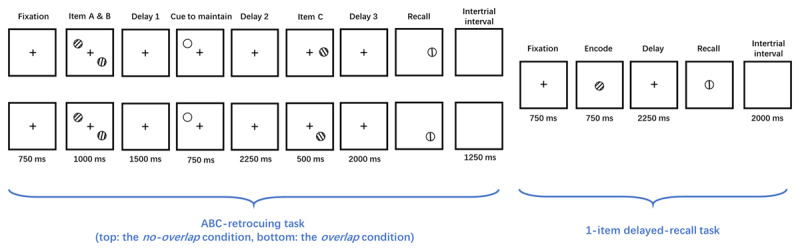
The experimental procedure. Each trial pairing comprises a trial of ABC retrocuing followed by a trial of 1-item delayed recall. In the ABC-retrocuing task, the presentation of two sample items (A and B) is followed by a retrocue, then by the presentation of a third sample item (C). The onset of the response wheel prompts recall of the orientation of either the retrocued item or item C. In the *no-overlap* condition (top) item C appears at a location different from that of the first two samples; in the *overlap* condition (bottom) C appears at the same location as had the IMI. In the linked 1-item delayed-recall task, both the sample and the response wheel appear centrally.

In a previous study (the “preliminary” study; Figure 1), we tested whether subjects use different strategies to remove information from WM under different conditions: passive loss when interference from a no-longer relevant item is expected to be low, versus active removal when interference is expected to be high. We did so with an “ABC” retrocuing procedure that began each trial with the simultaneous presentation of two Gabor patches, “item A” and “item B”, at two of a possible six locations. After a brief delay period a retrocue indicated which of the two might be tested at the end of the trial (for this explanation item A will always be the initially cued item). After a second delay period, a third stimulus (“item C”), was presented briefly. Finally, after a third delay period, a response wheel appeared at the location at which A or C had appeared, indicating which item was to be recalled. There were two conditions of ABC retrocuing: an *overlap* condition in which C always appeared at the location that had been occupied by B; and a *no-overlap* condition, in which C’s location was randomly selected from among the four locations that had been occupied by neither A nor B on that trial. Order of blocks was counterbalanced across subjects. The logic of the design was that trials in the *no-overlap* condition might promote the passive loss of the item that was designated the “irrelevant memory item” (IMI) by the retrocue (“item B” in this example), because minimal interference would be expected for the context cue provided by the location of the probe. (That is, even if a decaying representation of item B was still present in WM, a probe cuing the location of A or the location of C would be unlikely to trigger the retrieval of B.) Trials in the *overlap* condition, in contrast, would promote the active removal of the IMI, so as to minimize the “cue conflict” arising from one location being bound to both item B and item C (Lewis-Peacock et al., 2018).

In both conditions, each trial of ABC retrocuing was followed by a trial of 1-item delayed recall of orientation. Evidence for serial dependence of the recall of that single item on each of the three items from the preceding ABC-retrocuing trial would be taken as evidence for how those items had been processed. Of particular interest was the serial dependence of 1-item recall on the IMI (i.e., on item B), because an attractive influence of the IMI would suggest that it was not actively removed. Therefore, the absence of an attractive influence of the IMI from *overlap* blocks, paired with an attractive serial dependence on the IMI from *no-overlap* blocks, would be interpreted as evidence that the IMI had been actively removed from WM on *overlap* trials.

**Figure 2:**
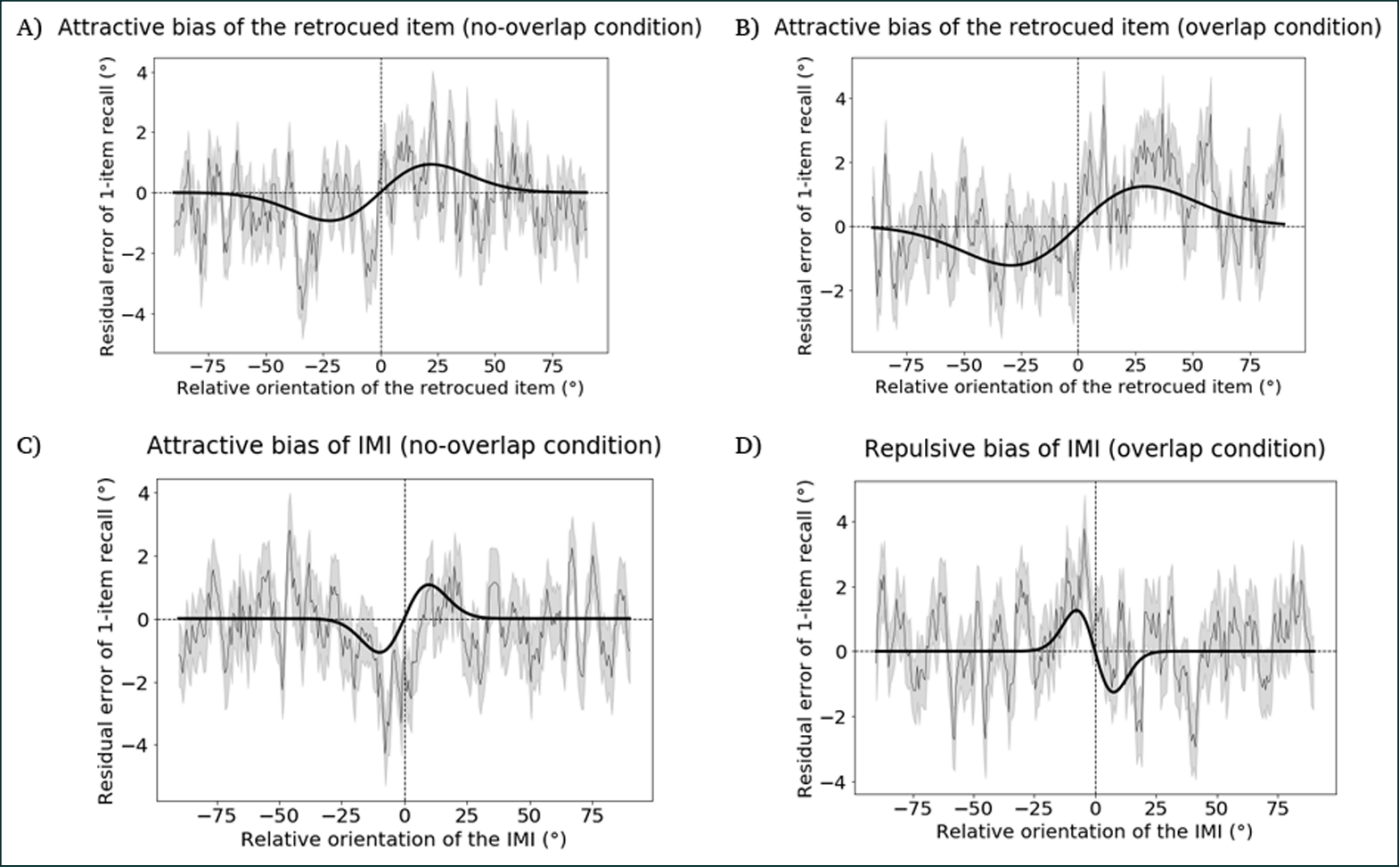
Results from the preliminary task, illustrating residual error of 1-item delayed recall as a function of angular difference with an item from the preceding ABC-retrocuing trial. **A.** Attractive bias of retrocued item in the *no-overlap* condition. **B.** Attractive bias of retrocued item in the *overlap* condition. **C.** Attractive bias of IMI in the *no-overlap* condition. **D.** Repulsive bias of IMI in the *overlap* condition. In each panel the thin black line represents the moving average of residual errors, with the SEMs in gray shading. The width of the moving average window is 100 datapoints and the step size is 20 datapoints. The thick black line shows the DoG fit to the data.

To test this prediction, for each trial pair we calculated the difference of the IMI orientation from the ABC-retrocuing trial to the target orientation in the linked 1-item delayed-recall trial, and used this value to predict the error of the report on 1-item delayed recall. The data were fitted with a derivative of Gaussian (DoG) tuning function for *overlap* and *no-overlap* conditions separately. Additionally, as sanity checks, we performed the same analyses of the influence of the retrocued item (i.e., “item A”) on 1-item delayed recall. The results, in reverse order of theoretical interest, were as follows. Results from the sanity checks confirmed the validity of our experimental logic, with the cued item exerting an attractive influence on linked 1-item recall in both the *no-overlap* (Figure 2A) and the *overlap* (Figure 2B) conditions. Next, an attractive serial dependence effect for the IMI in the *no-overlap* condition confirmed our expectation of a passive loss of this item in this condition (Figure 2C). We were surprised, however, by the results with the IMI in the *overlap* condition: Instead of the predicted null effect, the IMI produced a repulsive bias on the report from the linked 1-item task (Figure 2D). (That is, the recall of the sample on the 1-item trial was repelled away from the value of the IMI from the immediately preceding ABC retrocuing trial.) Although this outcome was broadly consistent with idea that the IMI had been processed differently on these trials than it had been on *no-overlap* trials, it did not fit with our expectations of how an active removal process would influence the serial bias of the IMI. From the perspective of the interference model, for example, a mechanism that acted by unbinding an item’s content from its context would be expected to produce either no serial bias (if that bias was due residual binding strength) or perhaps a weakened attractive bias (if the bias was due to the item’s activation strength, which might be expected to passively return to baseline upon being unbound). How, then, are we to understand this pattern from the preliminary study — a repulsive serial dependence effect for IMIs from *overlap* trials — and what implications might it have for our interest in the active control of information held in WM?

This Registered Report is motivated by reasoning that draws from two recent studies. In one, Fritsche et al. (2020) carried out analyses of serial dependence as a function of lag, on tests of 1-item recall that did not involve cuing. They observed a systematic flip in the sign of an item’s bias on subsequent behavior, from an attractive bias on the very next trial (which followed within just a few seconds) to a repulsive bias that was reliable beginning three trials later and that persisted for an additional six trials (covering many tens of seconds) or more, depending on specific task conditions. A series of analyses and simulations led to the conclusion that two factors were at play: Bayesian decoding that was responsible for the attractive bias, and an adaptation-like modification of perceptual circuitry (which they characterized as “efficient encoding”) that was responsible for the repulsive bias (Fritsche et al., 2020). Fritsche et al. (2020) implemented efficient encoding in their model via the reallocation of sensory encoding resources (in effect, biasing the way that subsequently presented stimuli would be processed). The second study motivating this Registered Report is an fMRI study of a variant the DSR task from Lorenc, Vandenbroucke et al. (2020). Of particular interest was the fate of the item that was designated the IMI by the retrocue. Multivariate pattern analysis (MVPA) indicated that evidence for an active representation of the IMI was negative relative to its representation prior to the retrocue. A follow-up analysis with multivariate inverted encoding modeling (IEM) produced a finding analogous to the MVPA – IEM reconstruction of the IMI was opposite to its reconstruction from earlier in the trial, when it was still potentially relevant (c.f., Sahan, Sheldon, & Postle, 2020). Follow-up simulations suggested that the most likely explanation for these results was that the gain of orientation-tuned sensory channels specific to the IMI had been modified. That is, the mechanism identified by Lorenc, Vandenbroucke et al. (2020), although deduced from very different data and with very different analytic methods, is conceptually very similar to the “efficient encoding” mechanism proposed by Fritsche et al. (2020). The potential significance of this observation is that it may point to a novel mechanism for the implementation of top-down control: Cue-induced active removal of an IMI (c.f., Lorenc, Vandenbroucke et al., 2020) may be implemented by a phasic “hijacking” of the same mechanisms that underlie adaptation of perceptual circuits (c.f., Fritsche et al., 2020), a process that is generally believed to result from the intrinsic response of sensory circuits to recent perceptual history (Clifford et al., 2000; Clifford et al., 2001). Because the *overlap* condition of the ABC retrocuing task from our preliminary experiment also involves the cue-induced removal of an IMI, replication of its finding of a repulsive serial bias in this condition would strengthen the proposition that the mechanisms proposed by Fritsche et al. (2020) and by Lorenc, Vandenbroucke et al. (2020) share common properties. This, in turn, would bolster the evidence for this novel mechanism for the active removal of information from WM.

With this Registered Report we are proposing to replicate the findings from the preliminary study: an attractive bias for the serial dependency of the IMI in the *no-overlap* condition, and a repulsive bias in the *overlap* condition. The empirical rationale for this replication study is two-fold: First, the repulsive bias we found in the *overlap* condition in the preliminary study was not predicted; second, due to a programming error, the orientation of item C in the ABC-retrocuing task was not recorded. Knowing the orientation of item C is important for ruling out the possibility that the apparent serial dependency effects of the IMI aren’t actually mediated through, and therefore attributable to, item C. By this alternative account, it is possible that the model’s estimate of the repulsive influence of the IMI is due to the IMI’s influence on item C (i.e., an interaction occurring within the ABC-retrocuing trial) rather than the IMI’s direct influence on the subsequent 1-item recall. Therefore, we compared a model assuming a direct effect of the IMI against a model assuming an indirect effect of the IMI that is mediated by item C. Thus, this Registered Report also helps clarify whether or not the apparent influence of the IMI on the linked 1-item delayed-recall task is actually due to the influence of the IMI on item C.

### Preregistered hypotheses

We propose to test 4 hypotheses in this Registered Report:

*Hypothesis 1*: The orientation of the retrocued item in the ABC-retrocuing task will significantly attract the report of the memory item on the subsequent 1-item delayed-recall task. This attractive effect will be present in both *overlap* and *no-overlap* conditions. (For this and the subsequent hypotheses, the precise method we will use to assess serial dependency is described in the section “Methods – Data analysis – Model fitting”.)

*Hypothesis 2*: In the *no-overlap* condition, the orientation of IMI in the ABC-retrocuing task will significantly attract the report of the memory item on the subsequent 1-item delayed-recall task.

*Hypothesis 3*: In the *overlap* condition, the orientation of the IMI in the ABC-retrocuing task will have a significant repulsive bias on the report of the memory item in the subsequent 1-item delayed-recall task.

*Hypothesis 4*: The error of linked 1-item delayed recall, when calculated as a function of the IMI, will be better explained by the orientation of the IMI than by the influence of the IMI on item C. (To be more specific, we will use two models to predict the error of recall in the 1-item delayed-recall task. In the “direct-influence” model, the error of the report will be predicted by the difference between the orientation of the IMI and that of the sample from the subsequent 1-item trial. In the “indirect influence” model, we will first model the influence of IMI on item C (i.e., a within-trial influence), and then predict the error of 1-item recall by the difference between the orientation of this “IMI-influenced item C” and that of the sample from the subsequent 1-item trial. Thus, *Hypothesis 4* holds that the fit of the direct-influence model, as measured by the Akaike Information Criterion, will be better than the fit of the indirect-influence model in both the *overlap* condition and the *no-overlap* condition. (See “Methods – Data analysis – Model fitting,” and “Methods – Data analysis – Model comparison” for precise details.))

## Methods

For each subsection in the Methods we will first describe the methods from the preliminary study, then the methods that we used for the Registered Report.

### Subjects

#### Preliminary study

One-hundred-and-twenty subjects were recruited remotely from Amazon Mechanical Turk with compensation of US $15. Subjects were given informed consent. They were identified as “Master Turk workers” older than 18 years of age, with lifetime approval rates no lower than 98%, and located the United States. All experimental procedures, including the acquiring of informed consent, were approved by the University of Wisconsin–Madison Health Sciences Institutional Review Board.

Upon completion of the task, subjects were debriefed about the strategy they used to remember Gabor patch orientation, and the data from those who reported using a nonvisual strategy (e.g., verbal [e.g., “I tried to think of it like a clock – 10:00, 11:00, 1:00, etc.”] or a motoric strategy [e.g., “I held my hands at what I thought were the right angles until it was time to report”, or “I moved my mouse in the direction of one patch”]) were removed from the analyses, as were the data from those who reported that they did not understand the task instructions. 20 subjects were removed for these reasons. For the remaining subjects, we calculated the average absolute recall error on the 1-item delayed-recall task, and the data from 4 subjects whose average absolute error was 2 SD higher than the mean value were also removed. Thus, the results reported here are from an N of 96.

#### Registered Report

Using the data from the preliminary study to do power analysis indicates that we should collect data from 208 subjects to achieve power of 0.96 to detect the effect predicted in *Hypothesis 3*, which is the hypothesis of principal theoretical importance. (See “Power analysis for the Registered Report” for details.) Enrollment proceeded iteratively until data from an N of 208 useable subjects had been acquired: Initially we enrolled subjects until collecting data from 208 who did not report using a nonvisual strategy, then we calculated the group-average absolute recall error on the 1-item delayed recall and removed any subjects whose average absolute error was 2 SD higher than the mean value; then we enrolled the additional number of subjects needed to achieve a final N of 208 subjects, none of whom reported using a nonvisual strategy, and none of whom had an average absolute error equal to or greater than the numerical cutoff corresponding to 2 SD larger than the mean from the first iteration. Because we encountered difficulty recruiting a sufficient number of subjects with the criteria from the preliminary study — Master Turk workers with lifetime approval rates no lower than 98% and located in the United States — partway through data collection for the Registered Report we changed inclusion criteria to lifetime approval rate of 95% and dropped the geographical restriction. All experimental procedures for the Registered Report, including the acquiring of informed consent, were approved by the University of Wisconsin–Madison Health Sciences Institutional Review Board.

### Stimuli and procedure

#### Preliminary study

The experiments were implemented with PsiTurk, a software package designed to interface with the Mechanical Turk website. Sample stimuli were black-and-white Gabor patches (radius = 70 pixels, contrast = 0.3, spatial frequency = 0.035 cycle/pixel; standard deviation for the Gaussian envelope = 27 pixels; phases were randomly chosen from 0, 0.2, 0.4, 0.6, and 0.8 circles). Retrocues were unfilled circles the same size as the samples. The response wheel was a circle of the same size that was unfilled apart from a rotatable bar corresponding to the diameter of the circle. In the ABC-retrocuing task, stimuli could appear in one of 6 locations centered on an imaginary circle with a radius of 300 pixels from central fixation and each spaced 60° distant from the nearest locations. In the 1-item delayed-recall task all stimuli were presented centrally.

The experiment consisted of 6 blocks of 20 trial pairs, each pair comprising a fixed sequence of one trial of ABC retrocuing followed by one trial of 1-item delayed recall (Figure 1). Each trial of ABC retrocuing began with a 750-msec fixation period and a subsequent simultaneous 1000-ms presentation of two samples (“item A” and “item B”), one at a location randomly selected from the 6 possible locations and the other at a location randomly selected from the remaining 5. After a 1500-ms delay period a retrocue appeared for 750 ms at the location where either A or B had appeared, indicating which of the two had a 50% likelihood of being tested and which one, by implication, was no longer relevant for that trial (i.e., the IMI). Retrocue offset was followed by a 2250-ms delay period, after which a third sample (“item C”), was presented for 1000 msec. In the *overlap* condition, item C always appeared at the location where the IMI had been presented, and in the *no-overlap* condition, it appeared in one of the four locations where neither A nor B had appeared on that trial. The offset of C was followed by 2000-ms delay period, after which the response wheel appeared, with equal probability, at the location where the retrocued item or where item C had been presented. Subjects were instructed to indicate the orientation of the sample item that had appeared at the probed location by rotating the dial with the computer mouse until it matched their memory of the probed sample item. There was no time limit, and the trial ended with a spacebar press to confirm the report. 1250 ms after the spacebar press the fixation for the linked trial of 1-item delayed recall was shown for 750 ms. Then, the sample stimulus appeared centrally for 750 ms, followed by a delay of 2250 ms. Recall of the sample was prompted by the central onset of the recall wheel, the subject’s response again confirmed with a spacebar press. The intertrial-pair interval was 2000 ms. Testing paused after each block, with the subject initiating every block with a spacebar press. On each trial of both tasks, sample orientation was selected randomly with replacement from a pool of every possible integer value from 0° to 179°.

Condition order was counterbalanced across subjects (three blocks of *overlap* first or three blocks of *no-overlap* first). Prior to the first block, subjects received verbal instruction and performed 4 practice trial pairs with the same condition as for the first three experimental blocks, which unfolded at a slower pace (longer stimulus presentations in the ABC-retrocuing task (of 2000 ms and 1500 ms) and a longer stimulus presentation in the 1-item delayed-recall trial (of 1750 ms)), before the 6 blocks of experimental tasks. Task instructions included an explanation of the overlap manipulation, and subjects were informed explicitly which condition was being tested prior to the first and the fourth block.

#### Registered Report

Stimuli and procedure in the Registered Report were identical to those used in the preliminary study.

### Data analysis

#### Preliminary study

##### Preprocessing

These steps preceded fitting the model to the data. First, data from all subjects were combined into one big dataset, and all analyses were conducted on this merged dataset. Next, trials in which the error of recall in the 1-item delayed-recall task was greater than 2 SD from the mean error were removed. (408 trials were removed from the overall total of 11, 520. Among the remaining 11,112 trials, 5,551 trials were from the *overlap* condition and 5,561 trials were from the *no-overlap* condition.) Lastly, we removed the systematic bias of recall from the 1-item delayed-recognition data by subtracting the mean response error, to obtain residual error.

##### Model fitting

We modeled the tuning of the serial-dependence effect to differences between the item of interest from each ABC-retrocuing trial (either the retrocued item or the IMI) and the sample of the linked 1-item delayed recall trial with the derivative of Gaussian (DoG). Following (Bliss et al., 2017), the DoG function is:

y = xawc{e^{ - {{\left({wx} \right)}^2}}}

where *y* is the residual error of recall from 1-item delayed recall, *x* is the angle of the retrocued item or the IMI relative to the sample of the linked 1-item delayed-recall trial, *a* is the amplitude of the curve peaks, *w* is the width of the curve, and *c* is the constant \sqrt 2 /{e^{ - 0.5}}.

We used the least_squares function in the scipy package to fit the model and found the optimal values of *a* and *w* that minimize the difference between *y* and \hat y for each combination of memory item (retrocued item or IMI) and condition (*overlap* or *no-overlap*) separately. To estimate the magnitude of the bias, we determined the peak-to-peak distance by calculating the difference between the maximum and the minimum of the prediction of the model, with the sign adjusted to match the direction of the bias (i.e., positive for attractive bias and negative for repulsive bias).

To determine whether influence of the retrocued item or the IMI on 1-item delayed recall was statistically significant, we did a permutation test for the effect of the retrocued item and of the IMI separately for each condition. We shuffled the x label (i.e., the angle of the retrocued item or the IMI relative to the 1-item sample) of the dataset and fit the DoG to this shuffled dataset. By doing so, we obtained a pair of parameter estimates for *a* and *w*. Then, we repeated this process for 1,000 times and got a distribution of values of *a* and *w*, separately. Because the dataset was shuffled, distributions of *a* and *w* were considered to be the null distributions under the H_0_: There is no serial dependence between the retrocued item/IMI and the linked sample. The *p*-values were the proportion of the null distribution that led to a more extreme peak-to-peak distance than the true peak-to-peak distance estimated from the original dataset.

To test whether the IMI had a different influence on 1-item recall under the two conditions, we did a permutation test in which we randomly relabeled the condition assignment of data points and calculated the difference of peak-to-peak distance between the *no-overlap* condition and the *overlap* condition, and repeated this 1,000 times. The proportion of differences of peak-to-peak distances in the relabeled datasets that was more extreme than the difference of peak-to-peak distances in the original dataset was the *p*-value for this permutation test.

We also computed bootstrapped confidence intervals of peak-to-peak distances separately for each combination of memory items and conditions. We resampled the data with replacement 1,000 times and fit the DoG to each resampled dataset. This yielded a distribution of peak-to-peak distances and we selected the 95% confidence interval from it.

#### Registered Report

##### Preprocessing

We used the same method as the preliminary study to preprocess the data.

##### Model fitting

We used the same methods as the preliminary study to fit the data, to statistically test the bias effect, and to calculate confidence intervals. We also carried out this analysis on the subset of trials in which the retrocued item (i.e., item “A”) is probed. This additional analysis acted as an “exploratory control” by assessing evidence that the predicted reversal of the serial bias of the IMI in the *overlap* condition wasn’t due to specific effects that the IMI may have on the recalled item C. We qualify it as “exploratory” because the statistical power of this Registered Report has been determined for the full data set, and halving the number of trials for this additional analysis may result in an underpowered analysis yielding equivocal results.

##### Model comparison

To assess the possibility that the apparent serial dependence of 1-item recall on the IMI might actually be mediated by item C, we also built an “indirect-influence” model, which entailed two steps. The first step was to estimate the WM representation of C biased by the IMI:

{item\_C}^{\prime}= x_{imi}{a_1}{w_1} ce^{-(w_{1}x)^{2}}+ item\_C

where *item_C*′ is the orientation of item C in WM biased by IMI, *item_C* is the true orientation of item C, *x*_*imi*_ is the angle of IMI relative to *item C, a*_1_ is the amplitude of the curve peaks, *w*_1_ is the width of the curve and c is the constant \sqrt 2 /{e^{ - 0.5}}. The second step of building the indirect-influence model was to estimate the serial dependence of 1-item delayed recall on *item_C*′:

y = {x_{{item\_C}^{\prime}}}{a_2}{w_2}c{e^{-{({{w_2}x})}^2}}

where *y* is the residual error of recall in the 1-item task, *x*_*item_C*__′_ is the angle of item_C’ relative to *the sample* in the linked 1-item task, *a*_2_ is the amplitude of the curve peaks, *w*_2_ is the width of the curve and c is the constant \sqrt 2 /{e^{ - 0.5}}.

We used the least_squares function in the scipy package to fit the indirect model and find the optimal values of *a*_1_, *w*_1_, *a*_2_, and *w*_2_ for each condition (*overlap* or *no-overlap*) separately.

We also built a null model in which the items in the ABC-retrocuing trial exert no influence on the recall of the sample in the linked 1-item trial, such that:

y = 0

where *y* is the residual error of recall in the 1-item task.

We used the Akaike Information Criterion (AIC) with the standard correction for finite sample sizes (AICc) to compare the direct-influence model of serial dependence on the IMI, as described in the “Model fitting” section, the indirect-influence model of serial dependence on the item C’ and the null model for each condition separately.

## Results

### Preliminary study

#### Precision of recall

On the ABC-retrocuing task, the mean of absolute error, across subjects, for the retrocued item was 17.938° (SD 8.172°. Splitting by condition, *M*_*overlap*_ = 18.711° ± 9.315°, *M*_*no-overlap*_ = 17.166° ± 7.755°), before dropping trial pairs with poor performance. Due to a programming error, the precision of recall of item C was not collected. On the 1-item delayed-recall task, the mean absolute error was 9.348° (SD 3.672°. Splitting by condition, *M*_*overlap*_ = 9.298° ± 3.940°, *M*_*no-overlap*_ = 9.399° ± 3.998°) before dropping trial pairs with poor performance.

After dropping trial pairs with poor performance, the mean of absolute error, across subjects, for the retrocued item was 17.849° (SD 7.991°). Splitting by condition, *M*_*overlap*_ = 18.713° ± 9.240°, *M*_*no-overlap*_ = 17.019° ± 7.521°). For the 1-item delayed-recall task, the mean absolute error was 7.928° (SD 2.899°). Splitting by condition, *M*_*overlap*_ = 7.934° ± 3.137°, *M*_*no-overlap*_ = 7.934° ± 2.850°) after dropping poor-performance trial pairs.

#### Serial dependence of 1-item recall

The retrocued item produced a marginally significant attractive bias in the *no-overlap* condition (*p* = 0.061, permutation test; peak-to-peak distance = 1.865°; bootstrapped 95% confidence interval = [1.114°,4.855°], Figure 2A), and a significant attractive bias in the *overlap* condition (*p* = 0.019, permutation test; peak-to-peak distance = 2.463°; bootstrapped 95% confidence interval = [1.580°, 3.892°], Figure 2B). The IMI also produced a significant attractive bias in the *no-overlap* condition (p = 0.049, permutation test; peak-to-peak distance = 2.148°; bootstrapped 95% confidence interval = [–0.777°, 3.756°], Figure 2C). In the *overlap* condition, in contrast, the IMI produced a significantly repulsive bias (p = 0.011, permutation test; peak-to-peak distance = –2.516°; bootstrapped 95% confidence interval = [–4.628°, –0.772°], Figure 2D). The difference of peak-to-peak distances between the *no-overlap* condition and the *overlap* condition was 4.665°, which was significantly different from 0 (p < 0.001, permutation test).

### Registered Report

#### Precision of recall

On the ABC-retrocuing task, the mean of absolute error, across subjects was 20.561° (SD 8.496°. Broken out by condition, *M*_*overlap*_ = 21.093° ± 8.628°, *M*_*no-overlap*_ = 20.030° ± 9.294°), before dropping trial pairs with poor performance. On the 1-item delayed-recall task, the mean absolute error was 12.275° (SD 6.287°. Broken out by condition, *M*_*overlap*_ = 12.045° ± 6.432°, *M*_*no-overlap*_ = 12.506° ± 6.692°) before dropping trial pairs with poor performance.

After dropping trial pairs with poor performance, the mean of absolute error, across subjects, for the ABC-retrocuing task was 20.541° (SD 8.547°). Splitting by condition, *M*_*overlap*_ = 21.019° ± 8.670°, *M*_*no-overlap*_ = 20.072° ± 9.321°). For the 1-item delayed-recall task, the mean absolute error was 10.207° (SD 5.587°). Splitting by condition, *M*_*overlap*_ = 10.050° ± 5.474°, *M*_*no-overlap*_ = 10.384° ± 5.985°) after dropping poor-performance trial pairs.

### Hypothesis 1

**Figure 3:**
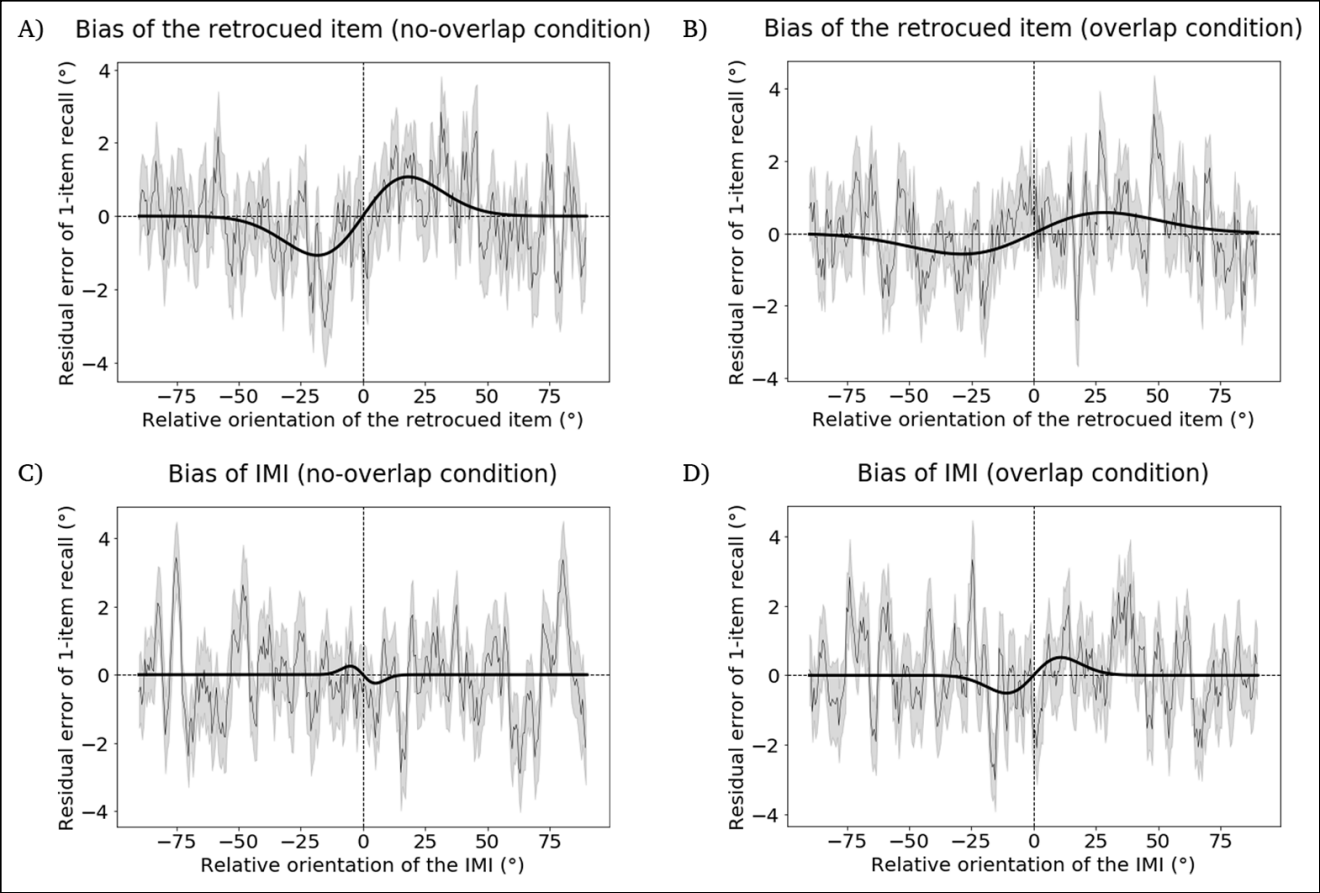
Results from the Registered Report, illustrating residual error of 1-item delayed recall as a function of angular difference with an item from the preceding ABC-retrocuing trial. **A.** Attractive bias of retrocued item in the *no-overlap* condition. **B.** The bias of retrocued item in the *overlap* condition. It is the absence of evidence for a significantly attractive bias in this condition that constitutes the failed sanity check. **C.** The bias of IMI in the *no-overlap* condition. **D.** The bias of IMI in the *overlap* condition. The width of the moving average window is 200 datapoints and the step size is 40 datapoints. Plotting conventions same as Figure 2.

In the *overlap* condition, the retrocued item failed to produce a significant attractive bias on 1-item delayed recall (*p* = 0.262, permutation test; peak-to-peak distance = 0.149°; bootstrapped 95% confidence interval = [–1.899°, 2.054°], Figure 3B), whereas in the *no-overlap* condition this effect was statistically significant (*p* = 0.034, permutation test; peak-to-peak distance = 2.144°; bootstrapped 95% confidence interval = [1.170°, 3.416°], Figure 3A). Note that the absence of an attractive serial-bias for the retrocued item in the *overlap* condition amounted to a failed sanity check, because interpretation of *Hypothesis 3*, the hypothesis of primary theoretical interest, would be uninterpretatble in the absence of the expected effect in this control condition. For completeness, however, we report the results of the remaining hypothesis tests carried out on these data.

### Hypothesis 2

*In the no-overlap condition*, the IMI did not produce the predicted attractive serial bias (p = 0.766, permutation test; peak-to-peak distance = –0.500°; bootstrapped 95% confidence interval = [–2.360°, 1.637°], Figure 3C).

### Hypothesis 3

In the *overlap* condition, the IMI did not produce the predicted attractive serial bias (p = 0.301, permutation test; peak-to-peak distance = 1.027°; bootstrapped 95% confidence interval = [–1.631°, 2.593°], Figure 3D).

The difference of peak-to-peak distances between the *no-overlap* condition (*Hypothesis 2*) and the *overlap* condition (*Hypothesis 3*) was –1.527°, which did not differ significantly from 0 (p = 0.302, permutation test).

#### Exploratory control

**Figure 4:**
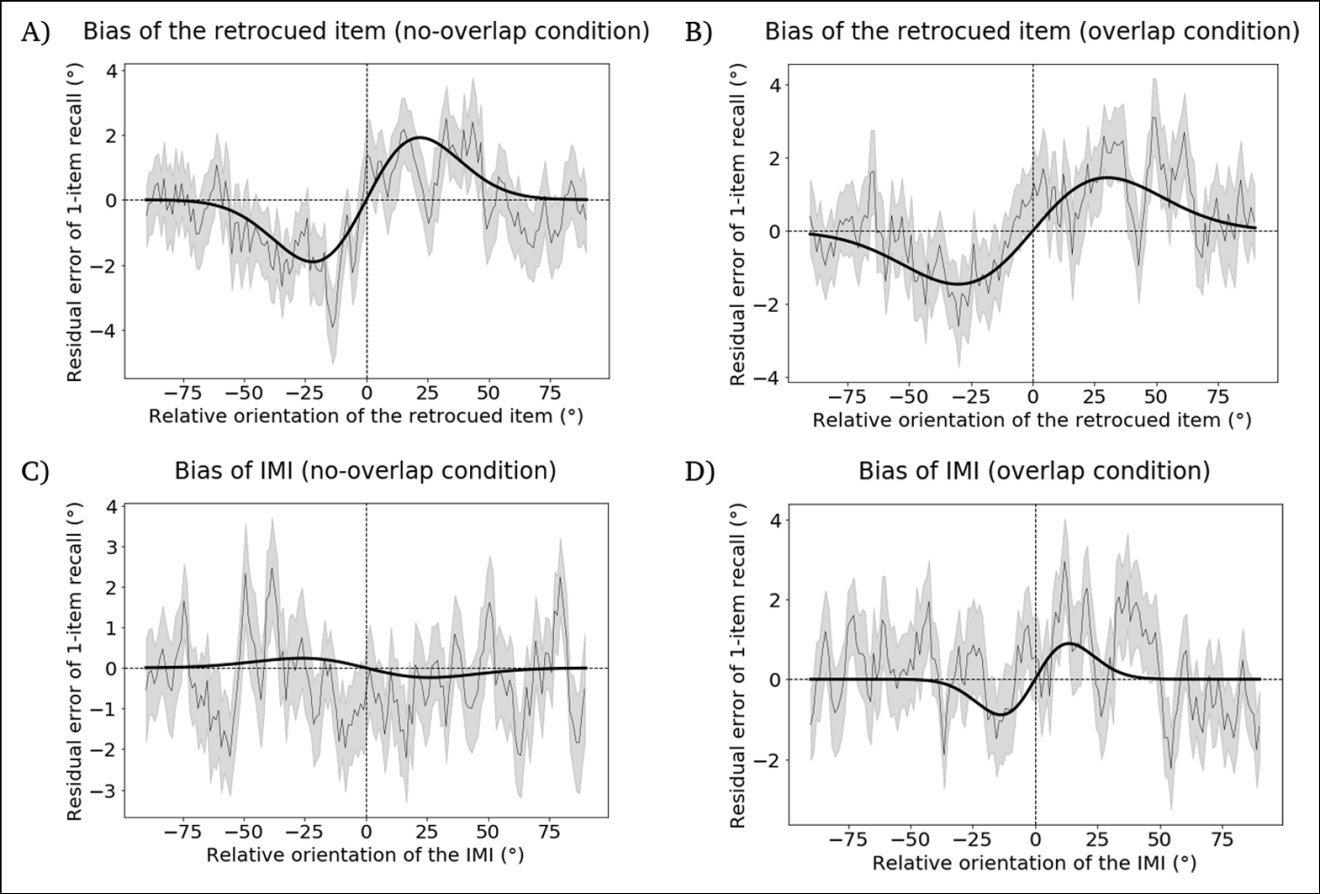
Results from the Registered Report, illustrating the bias on 1-item delayed recall induced by items from the preceding ABC-retrocuing trial, in the subset of trials in which item A was tested. **A.** Attractive bias of retrocued item in the *no-overlap* condition. **B.** Attractive bias of retrocued item in the *overlap* condition. **C.** The bias of IMI in the *no-overlap* condition. **D.** The bias of IMI in the *overlap* condition. The width of the moving average window is 200 datapoints and the step size is 40 datapoints. Plotting conventions same as Figure 2.

In the subset of trials in which the retrocued item (i.e., item A) was probed for report at the end of the ABC-retrocuing trial, we investigated the influence of the item A and the IMI on the report in the following trial. The retrocued item produced significant attractive biases in both the *no-overlap* condition (p = 0.008, permutation test; peak-to-peak distance = 3.822°; bootstrapped 95% confidence interval = [2.535°, 5.662°], Figure 4A) and the *overlap* condition (p = 0.025, permutation test; peak-to-peak distance = 2.913°; bootstrapped 95% confidence interval = [1.786°, 4.121°], Figure 4B). The IMI did not produce a bias in the *no-overlap* condition (p = 0.887, permutation test; peak-to-peak distance = –0.475°; bootstrapped 95% confidence interval = [–2.059°, 3.410°], Figure 4C) or the *overlap* condition (p = 0.226, permutation test; peak-to-peak distance = 1.738°; bootstrapped 95% confidence interval = [–2.461°, 4.009°], Figure 4D). The difference of peak-to-peak distances between the *no-overlap* condition and the *overlap* condition was –2.259°, which was not significantly different from 0 (p = 0.307, permutation test).

### Hypothesis 4

The indirect-influence model was the best among the three models in accounting the data for both the *no-overlap* condition and the *overlap* condition (*no-overlap* condition: ΔAICc _indirect-null_ = –34.713, ΔAICc _null-direct_ = –3.785; *overlap* condition: ΔAICc _indirect-null_ = –28.751, ΔAICc _null-direct_ = –1.695).

As noted above, the failure to observe, in the critical control condition, the attractive bias that is typically reported on reproduction-of-orientation tasks (e.g., Bliss et al., 2017; Fischer & Whitney, 2014; Fritsche et al., 2020) rendered the results of the test of *Hypothesis 3* uninterpretable. Although we cannot know definitively why the Registered Report failed to replicate the results from the preliminary study, which used the same procedures for online data collection, we note that the absolute report errors for 1-item trials were higher for the Registered Report than for the preliminary study: 10.207° ± 5.587° vs. 7.928° ± 2.899°, respectively). In order to generate interpretable data with this procedure, our next step was to avoid the lack of experimental control associated with online data collection (Woods et al., 2015) by carrying out a laboratory-based follow-up experiment that would employ, as closely as possible, the procedures from the Registered Report.

## Follow-up experiment

Because the failure to confirm *Hypothesis 1* of the Registered Report rendered the remaining results from the experiment equivocal, the authors undertook a follow-up experiment to retest the preregistered hypotheses, but with in-person testing procedures. This follow-up experiment was undertaken after obtaining approval from the editor who oversaw the peer review of the Registered Report.

### Methods

#### Subjects

Repeating the procedure of the Registered Report, but with in-laboratory data collection, allowed for enrolling 25 subjects (the same as the sample size used in experiments from Fritsche et al., 2020, which were also laboratory-based experiments). Subjects were recruited from the University of Wisconsin–Madison community and gave informed consent. Following the procedure from Registered Report, enrollment proceeded iteratively until data from an N of 25 useable subjects had been acquired: Initially we enrolled subjects until collecting data from 25 subjects, then we calculated the group-average absolute recall error on the 1-item delayed recall and removed any subjects whose average absolute error was 2 SD higher than the mean value; then we enrolled the additional number of subjects needed to achieve a final N of 25 subjects, none of whom had an average absolute error equal to or greater than the numerical cutoff corresponding to 2 SD larger than the mean from the first iteration. All experimental procedures, including the acquiring of informed consent, were approved by the University of Wisconsin–Madison Health Sciences Institutional Review Board.

#### Stimuli and procedure

Sample stimuli were black-and-white Gabor patches (radius = 3˚, contrast = 0.6, 1 cycle/˚, random phase angle) presented on gray background. Retrocues were unfilled circles the same size as the samples. The response wheel was a circle of the same size that was unfilled apart from a rotatable bar corresponding to the diameter of the circle. In the ABC-retrocuing task, stimuli could appear in one of 6 locations centered on an imaginary circle with a radius of 8° from central fixation and each spaced 60° distant from the nearest locations. In the 1-item delayed-recall task all stimuli were presented centrally.

Procedure in the follow-up experiment was identical to those used in the Registered Report except that each subject completed a total of 300 trial pairs for each condition across three sessions (with four 50-trial-pair blocks per session). Subjects did blocks of task under both conditions in each session with the first two blocks under one condition and the last two blocks under the other condition. Condition order was counterbalanced across sessions and across subjects.

#### Data analysis

We used the same method as the Registered Report to preprocess and analyze the data.

### Results

To achieve the final n of 25 (age = 21.4 ± 4.03, 14 females), 28 subjects were recruited and participated in portions of the experiment; two were not invited to complete all three sessions due to failure to comply with the instructions, and one was excluded from further analysis because of poor performance in the 1-item task. The first block of data from one subject was excluded because they reported not understanding the rules upon completion of this block.

#### Precision of recall

On the ABC-retrocuing task, the mean of absolute error, across subjects was 10.148° (SD 3.203°. Splitting by condition, *M*_*overlap*_ = 10.108° ± 3.309°, *M*_*no-overlap*_ = 10.195° ± 3.248°), before dropping trial pairs with poor performance. On the 1-item delayed-recall task, the mean absolute error was 6.227° (SD 1.839°. Splitting by condition, *M*_*overlap*_ = 6.152° ± 1.784°, *M*_*no-overlap*_ = 6.300° ± 1.936°) before dropping trial pairs with poor performance.

After dropping trial pairs with poor performance, the mean of absolute error, across subjects, for the ABC-retrocuing task was 10.056° (SD 3.197°). Splitting by condition, *M*_*overlap*_ = 10.018° ± 3.319°, *M*_*no-overlap*_ = 10.106° ± 3.241°). For the 1-item delayed-recall task, the mean absolute error was 5.333° (SD 1.367°). Splitting by condition, *M*_*overlap*_ = 5.283° ± 1.295°, *M*_*no-overlap*_ = 5.385° ± 1.453°) after dropping poor-performance trial pairs.

### Hypothesis 1

**Figure 5:**
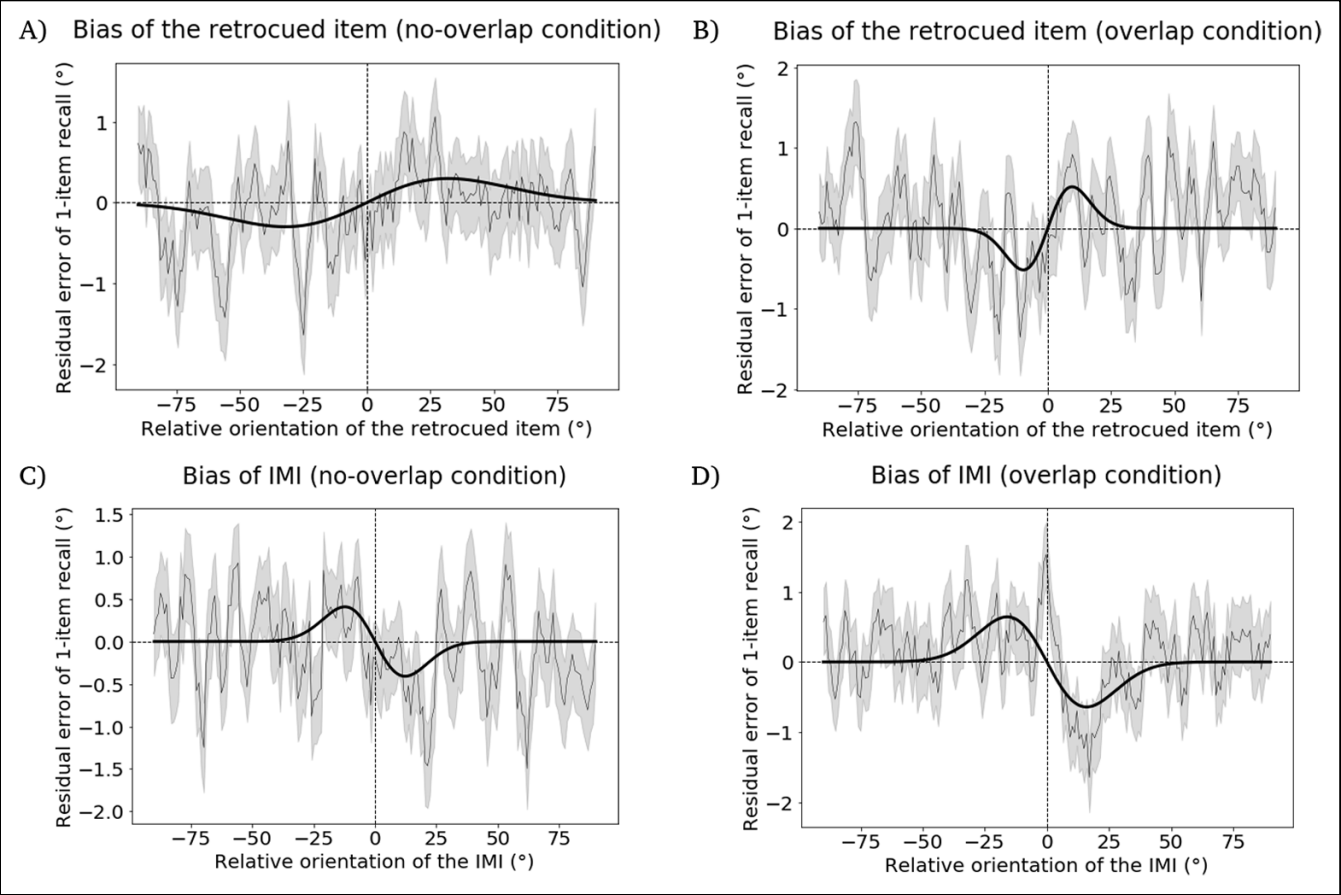
Results from the follow-up experiment, illustrating the bias on 1-item delayed recall induced by items from the preceding ABC-retrocuing trial. **A.** Bias of the retrocued item in the *no-overlap* condition. **B.** Attractive bias of the retrocued item in the *overlap* condition. **C.** Bias of the IMI in the *no-overlap* condition. **D.** Repulsive bias of the IMI in the *overlap* condition. The width of the moving average window is 200 datapoints and the step size is 40 datapoints. Plotting conventions same as Figure 2.

In the *no-overlap* condition the retrocued item produced a bias that was numerically attractive, but that did not achieve statistical significance (*p* = 0.34, permutation test; peak-to-peak distance = 0.597°; bootstrapped 95% confidence interval = [–0.678°, 1.506°], Figure 5A). In the *overlap* condition, the attractive bias approached significance (*p* = 0.081, permutation test; peak-to-peak distance = 1.032°; bootstrapped 95% confidence interval = [0.311°, 2.335°], Figure 5B). Although these results were weaker than hoped for, we judged the trend-level effect for the retrocued item in the *overlap* condition to be sufficient to support interpretation of *Hypothesis 3*, the hypothesis of primary theoretical importance for these experiments.

### Hypothesis 2

In the *no-overlap* condition, the IMI produced a numerically repulsive serial bias that did not achieve statistical significance (p = 0.188 permutation test; peak-to-peak distance = –0.818°; bootstrapped 95% confidence interval = [–1.650°, 0.471°], Figure 5C).

### Hypothesis 3

In the *overlap* condition, the IMI produced a significantly repulsive bias (p = 0.035, permutation test; peak-to-peak distance = –1.286°; bootstrapped 95% confidence interval = [–2.177°, –0.650°], Figure 5D).

The difference of peak-to-peak distances between serial dependence on the IMI in the *no-overlap* condition (*Hypothesis 2*) and in the *overlap* condition (*Hypothesis 3*) did not differ significantly from 0 (difference = 0.467°, p = 0.727, permutation test).

#### Exploratory control

**Figure 6:**
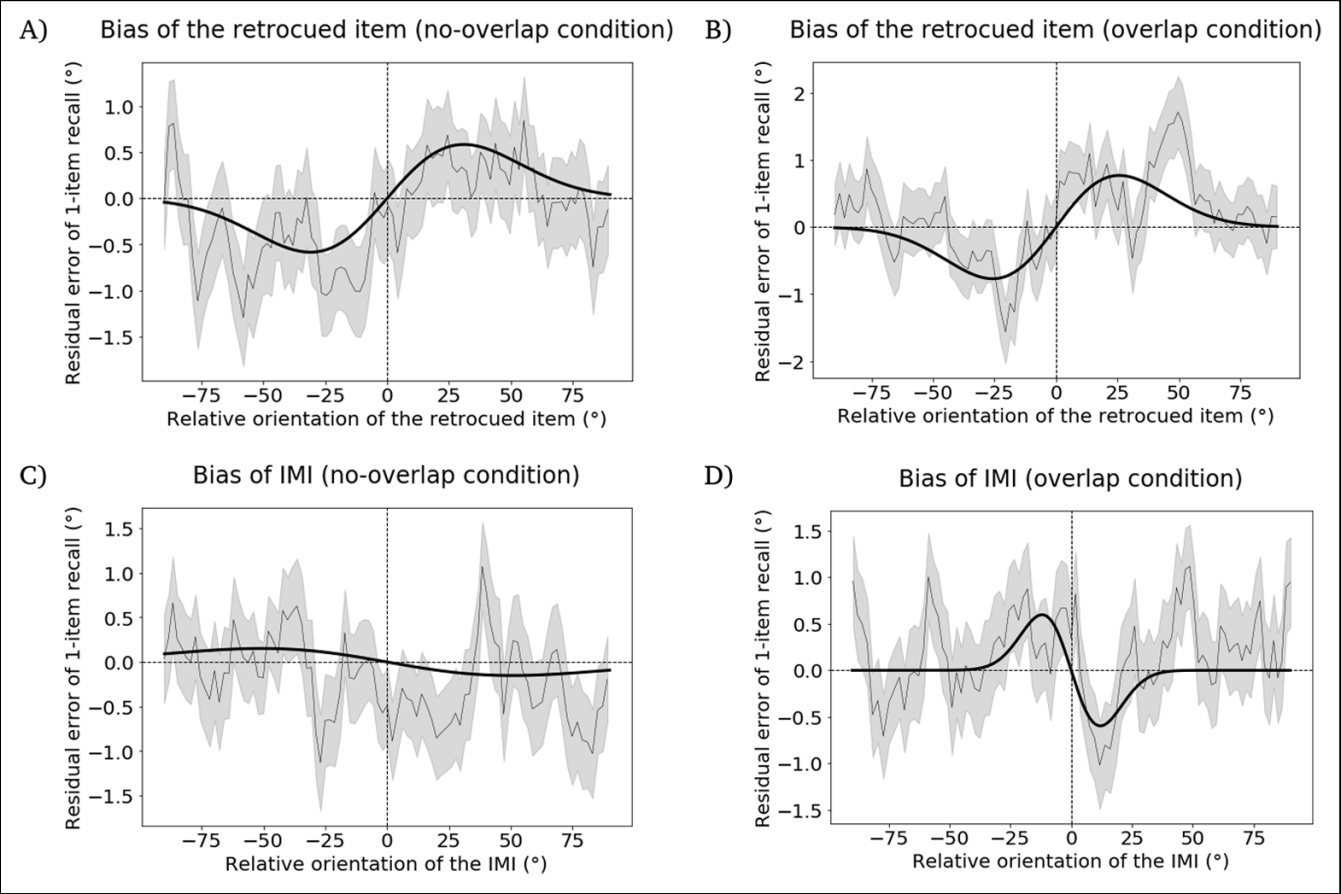
Results from the follow-up experiment, illustrating the bias on 1-item delayed recall induced by items from the preceding ABC-retrocuing trial, in the subset of trials in which item A was tested. **A.** Bias of the retrocued item in the *no-overlap* condition. **B.** Attractive bias of the retrocued item in the *overlap* condition. **C.** Bias of the IMI in the *no-overlap* condition. **D.** Bias of the IMI in the *overlap* condition. The width of the moving average window is 200 datapoints and the step size is 40 datapoints. Plotting conventions same as Figure 2.

As with the previous experiments, we also examined the subset of trials for which the retrocued item (i.e., item A) was probed for report at the end of the ABC-retrocuing trial. The retrocued item produced a nonsignificant attractive bias in the *no-overlap* condition (p = 0.175, permutation test; peak-to-peak distance = 1.169°; bootstrapped 95% confidence interval = [0.600°, 2.348°], Figure 6A) and a marginally significant attractive bias in the *overlap* condition (p = 0.058, permutation test; peak-to-peak distance = 1.543°; bootstrapped 95% confidence interval = [0.913°, 2.982°], Figure 6B). The IMI did not produce a bias in either the *no-overlap* condition (p = 0.878, permutation test; peak-to-peak distance = –0.304°; bootstrapped 95% confidence interval = [–1.792°, 1.072°], Figure 6C) or the *overlap* condition (p = 0.18, permutation test; peak-to-peak distance = –1.193°; bootstrapped 95% confidence interval = [–2.539°, 0.779°], Figure 6D). The difference of peak-to-peak distances between the *no-overlap* condition and the *overlap* condition was 0.889°, which was approached significant difference from 0 (p = 0.089, permutation test).

### Hypothesis 4

For both the *no-overlap* condition and the *overlap* condition, the indirect-influence model did the best job explaining the data, followed by the direct-influence model. The null model was the worst among the three (*no-overlap* condition: ΔAICc _indirect-direct_ = –24.159, ΔAICc _direct-null_ = –0.095; *overlap* condition: ΔAICc _indirect-direct_ = –11.671, ΔAICc _direct-null_ = –10.195). In the *overlap* condition, the parameter estimates of the indirect-influence model were *a*_1_ = –0.315, *w*_1_ = 2.452, *a*_2_ = 0.015, *w*_2_ = 2.239. In the *no-overlap* condition, the parameter estimates of the indirect-influence model were *a*_1_ = –1.299, *w*_1_ = 8, *a*_2_ = 0.016, *w*_2_ = 2.119. *a*_1_ indexes the sign (i.e., attractive vs. repulsive) and the amplitude of the influence of the IMI on item C, *w*_1_ determines the width of the fit of the DOG. *a*_2_ and *w*_2_ index the amplitude and width of the DOG corresponding to the influence of item C on the following 1-item trial. Thus, the negative *a*_1_ estimates suggest that the IMI repulsively biased item C in both conditions.

#### Exploratory analysis related to Hypothesis 4

**Figure 7:**
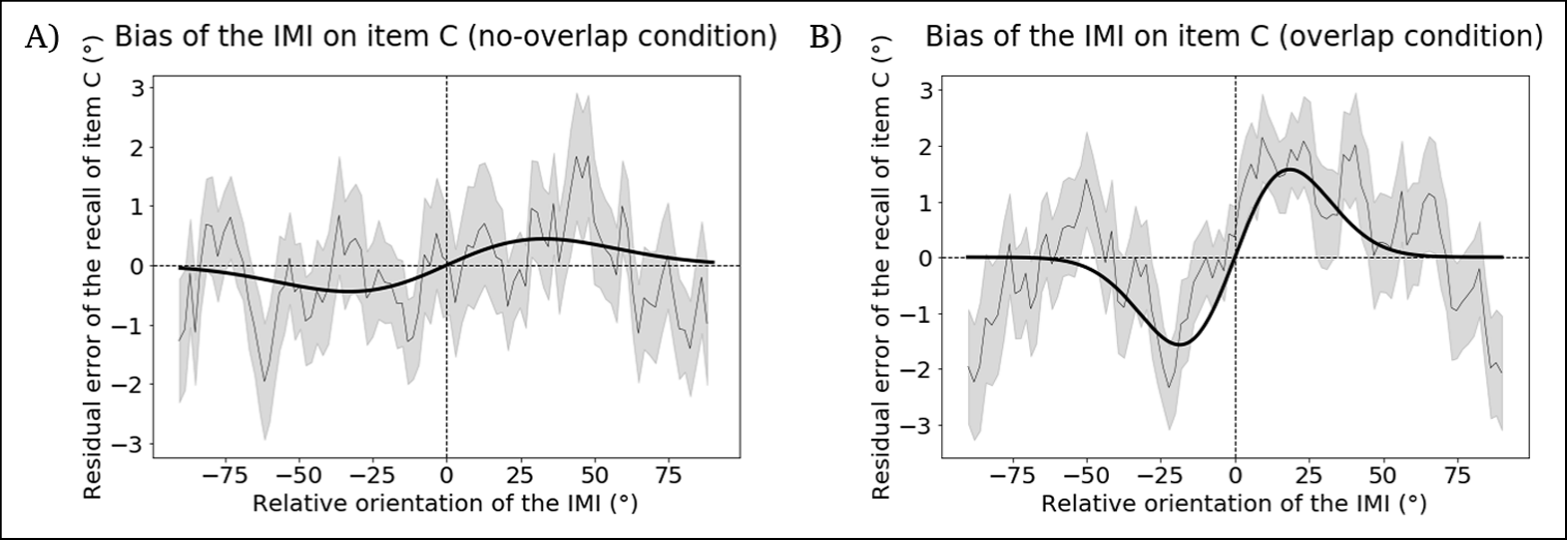
Results from the follow-up experiment, illustrating the bias on recall of item C induced by the IMI in the same trial, in the subset of trials in which item C was tested. **A.** Bias of the IMI in the *no-overlap* condition. **B.** Attractive bias of the IMI in the *overlap* condition. The width of the moving average window is 200 datapoints and the step size is 40 datapoints. Plotting conventions same as Figure 2.

Although the confirmation of *Hypothesis 3* means that the predicted pattern of a repulsive serial bias was observed in the follow-up study, the failure to confirm *Hypothesis 4* raises uncertainty about whether this effect was exerted by the IMI, directly, or indirectly via the IMI’s within-trial influence on item C. Thus, to assess the validity of the indirect-influence model, we carried out an exploratory analysis (i.e., not preregistered) in which we simply determined empirically whether the within-trial influence of the IMI on item C was repulsive or attractive, by examining the subset of trials for which item C was probed for report at the end of the ABC-retrocuing trial. The fact that the results reported for *Hypothesis 4* revealed negative values for the *a*_1_ parameter of the indirect model means that this model indicated a repulsive influence of the IMI on item C. Direct assessment of responses on trials when item C was recalled, however, indicated that the IMI produced a significantly attractive bias on item C in the *overlap* condition (p = 0.037, permutation test; peak-to-peak distance = 3.145°, Figure 7B), as well as a statistically nonsignificant but numerically attractive bias on item C in the *no-overlap condition* (p = 0.675, permutation test; peak-to-peak distance = 0.889°, Figure 7A). This invalidation of the indirect-influence model by the empirical data leaves the direct-influence model the model providing the best explanation of the results.

## Discussion

Interpreting the results of this collection of experiments is complicated by the failure of either the Registered Report or the follow-up experiment to fully replicate the pattern of results from the preliminary study. The Registered Report failed to meet a critical sanity check, in that the attractive serial bias that is typically observed with reproduction-of-orientation (e.g., Bliss et al., 2017; Fischer & Whitney, 2014; Fritsche et al., 2020; Samaha et al., 2019) was not produced by the cued item during *overlap* blocks. This rendered the serial bias of the uncued item (i.e., the IMI) in that condition — the effect of primary theoretical interest — uninterpretable. Consideration of the outcome of *Hypothesis 3* from the follow-up laboratory-based experiment, in isolation, suggests confirmation of the prediction that the serial biases exerted by the cued and uncued items in the overlap condition would be opposite in sign, and significantly repulsive for the IMI. This pattern of results, in isolation, is consistent with the view that subjects actively removed the IMI. This conclusion, however, is complicated by the fact that the serial bias exerted by the IMI in the *no-overlap* condition of this follow-up experiment was also numerically repulsive, albeit not at a level reaching statistical significance. (That is, *Hypothesis 2* was not confirmed, rendering the repulsive bias of the *overlap* IMI less specific than it had been in the preliminary study.)

Why were the serial bias effects reported here less stable across experiments than might have been predicted from the previous literature? One possibility is that the majority of experiments reporting an attractive bias of the current item on the subsequent trial feature a single to-be-remembered sample on each trial (e.g., Bliss et al., 2017; Fischer & Whitney, 2014; Fritsche et al., 2020; Samaha at al., 2019). Our ABC-retrocuing task, in contrast, was more complicated, presenting a total of 3 items per trial, and requiring an update of WM partway through the trial. A second, and mutually compatible explanation, is that our assumption that the overlap manipulation would generate two distinct strategies – active removal of the IMI during *overlap* blocks or passive loss during *no-overlap* blocks – may not have held. Our design did not include independent confirmation of strategy, and so we cannot rule out the possibility that, to varying degrees, subjects may have also engaged in active removal during *no-overlap* blocks, particularly during the follow-up experiment.

For Hypothesis 4, comparing three models, in both conditions the indirect-influence model worked the best, followed by the direct-influence model and the null model. However, a direct assessment of the empirical influence of IMI on item C clearly refutes the validity of indirect-influence model, by showing the influence of the IMI on item C was attractive, a finding opposite in sign to the prediction of the model. Thus, indirect influence of the IMI is not a tenable interpretation of the serial-dependence results from the follow-up experiment.

The serial dependence effect of the memory item from any given trial on the report of the subsequent trial, as typically found in previous studies, is attractive (e.g., Bliss et al., 2017; Fischer & Whitney, 2014; Fritsche et al., 2020; Samaha at al., 2019). Thus, the repulsive bias of the IMI from our ABC-retrocuing task on the recall of the subsequent 1-item delayed recall task is consistent with our assertion that this item was removed from WM in a manner fundamentally different from the cued item, which is assumed to have undergone “passive loss” after the end of the trial. With regard to the mechanism behind this active removal, our data are inconsistent with several existing accounts. In bump-attractor models, removal is accomplished by swamping and saturating the residual stimulus-representing synaptic traces (Barbosa, Stein et al. 2020), a mechanism that shouldn’t generate a repulsive serial bias. In the interference model, active removal is accomplished by breaking the item-context association (Lewis-Peacock et al., 2018), also an operation that is not expected to produce a repulsive serial bias. The proposition that we advance here is that the repulsive bias exerted by the IMI, as demonstrated in the results of the preliminary study and the follow-up study reported here, results from a rapid hijacking of the mechanisms of perceptual adaptation so as to suppress the active representation of this to-be-removed item.

## Power analysis for the Registered Report

To our knowledge, there is no established way to perform power analysis for permutation tests. In general, the statistical power of an experimental design is the probability that it would detect a true effect of a given effect size. In other words, if we could conduct an experiment many times, the power would correspond to the proportion of iterations for which a statistical test for this effect was significant. It has been shown that bootstrap resampling procedures can be used in power analysis to simulate data that are collected from future experiments without relying on the assumptions of the distribution of the data (Kleinman & Huang, 2016). Thus, bootstrapped resampling could be used to generate a simulated dataset, on which we could carry out a permutation test. The proportion of the simulated datasets that yield a significant outcome would correspond to the power of the experimental procedure. Thus, we retrospectively calculated the power of the preliminary study as follows. For the repulsive bias of the IMI in the *overlap* condition, which is predicted in *Hypothesis 3* of this Registered Report, we resampled, with replacement, the data in the *overlap* condition to create a new dataset of 5,551 data points (the same as the number of trials in the *overlap* condition in the original dataset) and did a permutation test of the influence of IMI on the recall of the linked sample in the 1-item task, as we did in “Method – modeling fitting,” but with only 100 reshuffles, to obtain a *p*-value for this resampled dataset. This process was repeated 100 times. The proportion of *p*-values that were smaller than 0.05 was .6, which corresponds to the power with *α* = 0.05. Using the same procedure to calculate the power of the attractive bias of IMI in the *no-overlap* condition, but with 5,561 data points (corresponding to the number of trials in the *no-overlap* condition in the original dataset), yielded a value of 0.57. Finally, for the bias caused by the retrocued item, power was estimated to be 0.79 and 0.62 in the *overlap* and *no-overlap* conditions, respectively.

For the prospective power analysis, we estimated the number of trials we would need to achieve power estimates greater than .9 by bootstrapping numbers of samples larger than what was the preliminary dataset (Kleinman & Huang, 2016). For the repulsive bias of IMI in the *overlap* condition, we resampled the data in the *overlap* condition from the preliminary study, with replacement, to create a new dataset of 12,000 data points, then carried out a permutation test to obtain *p*-value, and repeated this process 100 times. This yielded an estimated power of 0.96. Using the analogous method, we estimated that 12,000 data points would yield a power .93 to detect the attractive bias of IMI in the *no-overlap* condition, of 1.00 to detect the attractive bias of the retrocued item in the *overlap* condition, and of .96 to detect the attractive bias of the retrocued item in the *no-overlap* condition. Thus, to amass the 12,000 trials needed achieve these levels of power, we plan to enroll 208 subjects for the Registered Report.

## Data Accessibility Statement

All data and all codes for generating the tasks and conducting the analyses are available at https://github.com/ShanJG/active_removal_serial_dependence.
